# Multimodal neuroimaging protocol to explore the neural mechanisms of Tiao Shen Li Yan acupuncture in post-stroke dysphagia: a randomized sham-controlled clinical trial

**DOI:** 10.3389/fneur.2026.1764500

**Published:** 2026-06-19

**Authors:** Jing Luo, Yan Huang, Lijun Wang, Guogao Zhang, Shaoyang Cui, Wenke Tang, Xueping Ding, Xueying Zhao, Yiqin Huang, Zhenhua Xu, Zhentao Zuo

**Affiliations:** 1 The Second Clinical College of Guangzhou University of Chinese Medicine, Guangzhou, China; 2 Shenzhen Hospital (Futian) of Guangzhou University of Chinese Medicine, Shenzhen, China; 3 Peking University Shenzhen Hospital, Shenzhen, China; 4 MR Research, GE Healthcare, Beijing, China; 5 National Key Laboratory of Cognitive Science and Mental Health, Institute of Biophysics, Chinese Academy of Sciences, Beijing, China; 6 University of Chinese Academy of Sciences, Beijing, China

**Keywords:** acupuncture, diffusion tensor imaging (DTI), ischemic stroke, post-stroke dysphagia, randomized sham-controlled trial, resting-state functional MRI (RS-fMRI), structure-function coupling

## Abstract

**Background:**

Post-stroke dysphagia (PSD) remains a frequent and disabling sequela of ischemic stroke, and effective pharmacotherapy is still lacking. Acupuncture has demonstrated significant efficacy in improving swallowing function, yet its neural mechanisms remain unclear. Research into the functional reorganization of brain networks following acupuncture treatment remains insufficient.

**Objective:**

This protocol describes a randomized, single-blind, sham-controlled trial designed to evaluate the clinical effects and central neural mechanisms of Tiao Shen Li Yan acupuncture for PSD, using multimodal neuroimaging with resting-state functional MRI (rs-fMRI) and diffusion tensor imaging (DTI).

**Methods:**

Forty-six PSD patients will be randomized 1:1 to active or sham acupuncture (30 minutes sessions, 5 times per week for 6 weeks). The primary outcomes are rs-fMRI and DTI metrics reflecting functional and structural connectivity changes (ALFF, ReHo, FC, FA, MD). Secondary outcomes include Standardized Swallowing Assessment (SSA), videofluoroscopic swallowing study (VFSS)-based measures, Montreal Cognitive Assessment (MoCA), and Swallowing Quality-of-Life Questionnaire (SWAL-QOL). Twenty healthy volunteers will provide baseline neuroimaging reference data.

**Expected Results:**

The study aims to clarify how Tiao Shen Li Yan acupuncture modulates structure-function coupling in swallowing-related brain networks and promotes functional reorganization of the brain, and to determine whether these imaging biomarkers correlate with clinical improvement.

**Discussion:**

By integrating clinical assessments with multimodal neuroimaging under a rigorous randomized design and by incorporating healthy controls to identify abnormal brain network sites in PSD, this study predefines structure-function coupling metrics and clinical endpoint measures to further explore the neural mechanisms of brain network reorganization underlying acupuncture treatment for PSD. This protocol may provide mechanistic evidence for acupuncture-induced neuroplasticity and support the development of treatment strategies for PSD.

**Trial registration:**

https://www.chictr.org.cn/hvshowproject.html?id=286791&v=1.1, identifier: ChiCTR2400086748.

## 1 Introduction

In China, approximately 80% of strokes are ischaemic strokes, which constitute the primary cause of disability and death among adults (1). Dysphagia occurs in 21% to 78% of stroke patients and is associated with aspiration pneumonia and poor functional outcomes (2–4). Despite its clinical relevance, current rehabilitation approaches, such as swallowing exercises, physical therapy, and compensatory maneuvers, offer limited efficacy, and no targeted pharmacological treatment is available (5). Therefore, developing evidence-based, mechanism-informed interventions for PSD remains a critical unmet need in neurorehabilitation.

Acupuncture has been widely applied in post-stroke rehabilitation and has shown encouraging effects in improving swallowing function, with good safety and patient tolerance (5–7). However, the central neural mechanisms underlying its therapeutic effects remain poorly characterized. Traditional Chinese Medicine (TCM) attributes PSD to the “dysfunction of the Yuan-Shen” (primordial spirit), leading to “impaired throat function”. Guided by this theoretical framework, the Tiao Shen Li Yan acupuncture method regulates both systemic and local pathways by stimulating acupoints along the Governing (Du) [e.g., Baihui (GV20), Shenting (GV24)] and Conception (Ren) Vessel [e.g., Lianquan (CV23), Wangu (GB12), Fengchi (GB20), Tianzhu (BL10)]. Preliminary clinical observations from our group have demonstrated improved swallowing performance following this intervention (8), but objective neurophysiological evidence remains limited.

Recent advances in neuroimaging provide powerful tools to explore acupuncture-induced neuroplasticity (9). The spontaneous neural activity and functional connectivity of the brain's internal network can be assessed by resting-state functional MRI (rs-fMRI), and white matter connectivity and its microstructure can be assessed by diffusion tensor imaging (DTI). However, research on the mechanisms of brain functional network remodeling underlying acupuncture treatment for PSD is still insufficient. Rs-fMRI quantifies intrinsic activity (e.g., ALFF, ReHo) and functional connectivity to track network-level functional reorganization after stroke (10, 11). DTI evaluates white-matter microstructure (e.g., FA, MD) and can delineate structural disconnections within swallowing networks (12, 13). In post-stroke dysphagia, neural recovery is thought to involve both functional reorganization of swallowing-related networks and structural remodeling of white-matter pathways. Thus, investigating the interaction between structural and functional networks may provide insights beyond analyses of structural or functional alterations alone (14). Previous imaging studies of PSD have not focused on the interaction between functional and structural remodeling in swallowing-related networks, but only on changes in local activation.

To address this gap, we designed a single-blind, randomized, sham-controlled trial to elucidate the clinical efficacy and neural mechanism of Tiao Shen Li Yan acupuncture in treating PSD. By prespecifying multimodal neuroimaging endpoints—namely, rs-fMRI and DTI—the study aims to characterize how acupuncture modulates brain network organization during swallowing recovery. Specifically, we hypothesize that Tiao Shen Li Yan acupuncture enhances integration within swallowing-related functional networks, promotes microstructural repair of white-matter pathways, and strengthens structure-function coupling, thereby facilitating functional reorganization of brain networks and ultimately improving swallowing function and quality of life in patients with PSD.

This protocol adheres to the SPIRIT 2013 Statement and CONSORT guidelines for clinical trial design and reporting. The findings are expected to provide mechanistic evidence for acupuncture-induced neuroplasticity and to support the development of standardized, evidence-based rehabilitation strategies for PSD.

## 2 Methods and analysis

### 2.1 Registration and trial design

This single-blind, randomized, sham-controlled trial aims to investigate the therapeutic efficacy and neural mechanisms of Tiao Shen Li Yan acupuncture in patients with PSD.

**Figure 1:**
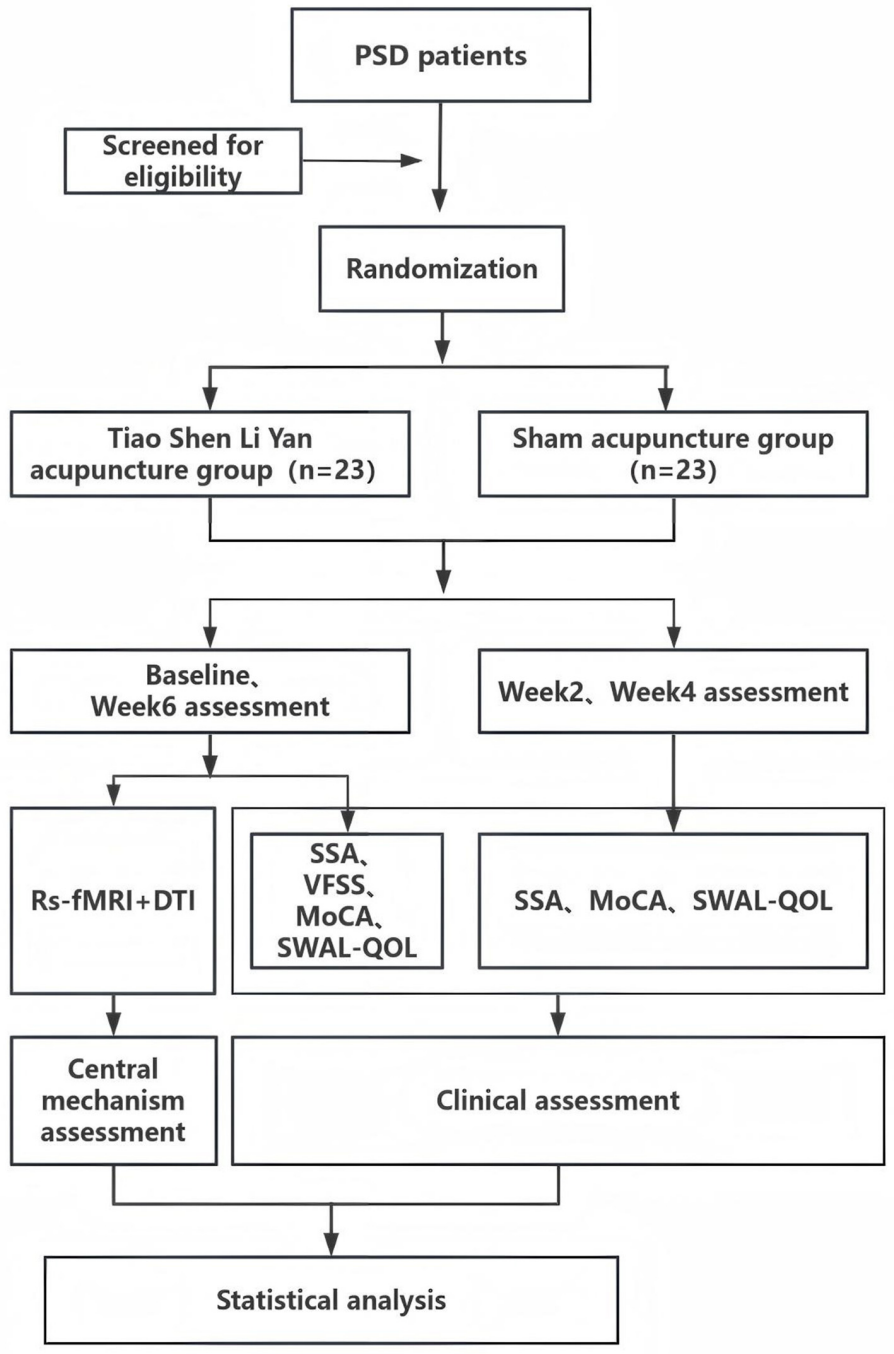
Flow chart of the clinical trial procedures.

Forty-six participants will be randomized in a 1:1 ratio to receive Tiao Shen Li Yan acupuncture or sham acupuncture. An additional cohort of healthy volunteers will undergo baseline rs-fMRI and DTI for reference comparisons. Participants will be included only if they fulfill all eligibility criteria and submit written informed consent. Figure 1 shows the research process for this article.

The trial has been prospectively registered in the Chinese Clinical Trial Registry (ChiCTR2400086748) on 10 July 2024. The trial registration was updated on 30 October 2025 to clarify the outcome hierarchy (rs-fMRI/DTI as the primary outcome; SSA/VFSS-based measures/MoCA/SWAL-QOL as secondary). No other changes were made.

### 2.2 Study setting and recruitment

The study will be conducted at Shenzhen Hospital (Futian) of Guangzhou University of Traditional Chinese Medicine. Recruitment will occur across inpatient and outpatient departments via notices and clinician referrals. Baseline demographics and clinical variables will be collected and stored in a secure database under the oversight of the Data Monitoring Committee (DMC), and will remain inaccessible to any person or institution not directly related to this trial.

The study protocol follows the SPIRIT 2013 statement and CONSORT guidelines for interventional clinical trials. Ethical approval has been granted by the Medical Ethics Committee of Shenzhen Hospital (Futian) of Guangzhou University of Chinese Medicine (original approval No. GZYLL (KY)-2023-052; amendment opinion No. GZYLL (KY)-2023-052-02).

### 2.3 Eligibility criteria

**Inclusion criteria:** (1) ischemic stroke and dysphagia diagnosed per accepted criteria (15, 16), with Kubota Water Swallowing Test (WST) ≥grade II; (2) aged 30–80 years; (3) stroke onset 2 weeks to 6 months prior; (4) conscious, hemodynamically stable, and able to comply with procedures; (5) written informed consent.

**Exclusion criteria:** (1) coma, Parkinson's disease, or Alzheimer's disease (AD); (2) Severe cardiac, renal, or other organ failure, or critically ill stroke with unstable vital signs. (3) failure to meet the diagnostic criteria for PSD or absence of cerebrovascular disease on neuroimaging; (4) inability to cooperate with evaluation or treatment, or poor adherence; (5) severe psychiatric disorders precluding cooperation with observation or treatment; and (6) contraindications to MRI (e.g., ferromagnetic implants).

### 2.4 Randomization and blinding

Randomization will be performed using simple randomization with a random number table. Randomization codes will be sealed in opaque, sequentially numbered envelopes, each containing the corresponding allocation card. Eligible participants will be enrolled in chronological order, and the envelopes will be opened sequentially. A study coordinator will assign participant identification codes and record them in the case report forms. The treating acupuncturists will open the envelopes in sequence and allocate participants accordingly. Baseline details (including name, sex, age, and enrollment date) will be recorded prior to randomization. Opened envelopes will be stored securely in a locked cabinet.

Given the nature of acupuncture, blinding of the acupuncturists cannot be fully implemented. However, outcome assessors, imaging analysts, data managers, and statisticians will remain blinded to allocation in order to minimize potential bias. Blinding procedures will be monitored by the institutional ethics committee. Unblinding will occur only after database lock and completion of primary analyses.

### 2.5 Interventions

#### 2.5.1 Tiao Shen Li Yan acupuncture group

**Figure 2:**
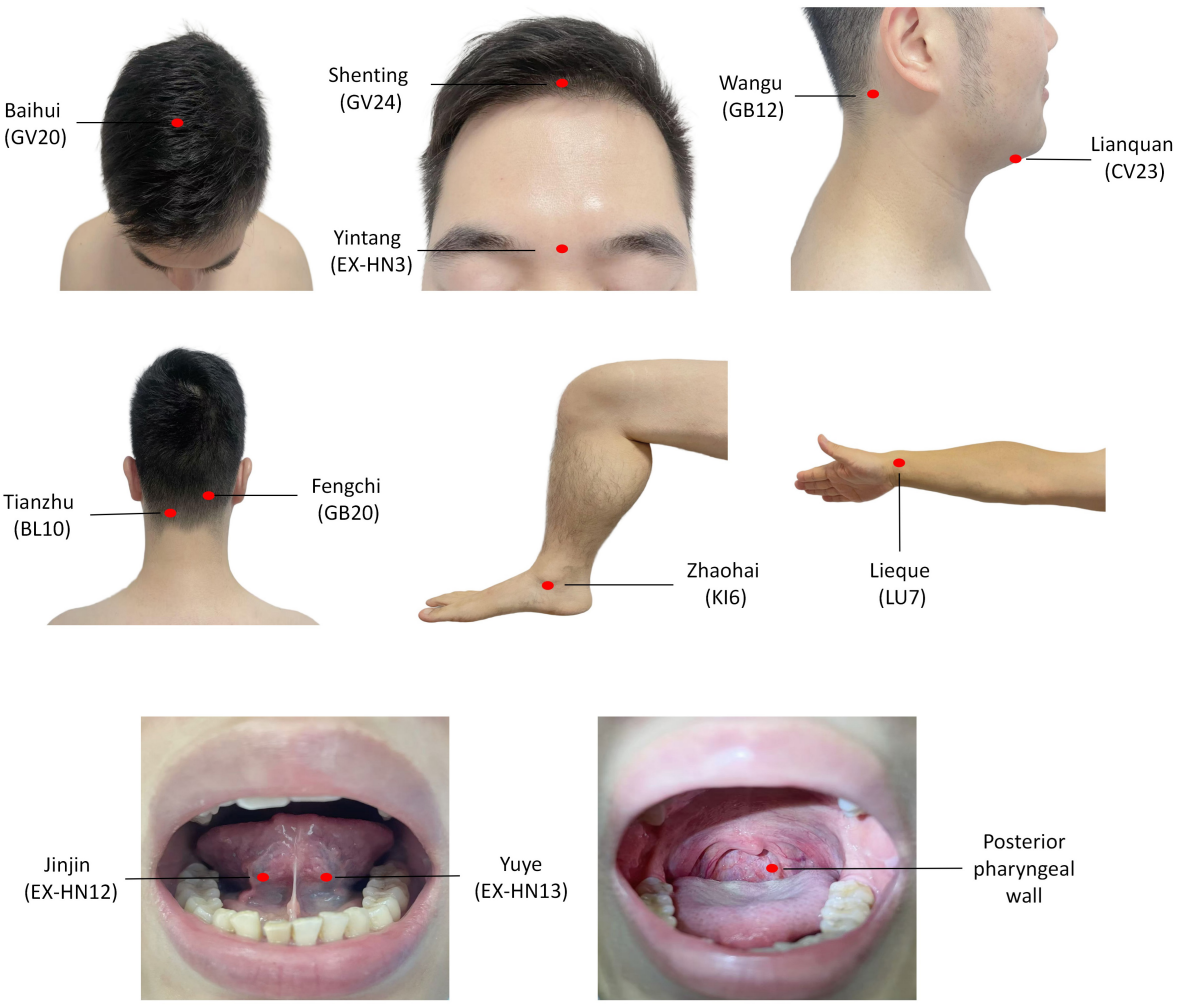
Acupoint locations for the Tiao Shen Li Yan acupuncture protocol. Acupoint Location for the Tiao Shen Li Yan acupuncture: Baihui (GV20): Located at the vertex on the midline of the head, where it crosses the line joining the apices of both ears; Shenting (GV24): Located 0.5 cun above the midpoint of the anterior hairline; Yintang (EX-HN3): Located in the middle between the ends of the eyebrows in the glabellar area; Lianquan (CV23): With the head slightly extended, the point lies above the Adam's apple, at the midpoint of the upper border of the hyoid bone body; Fengchi (GB20): Found below the occipital bone, in the depression between the sternocleidomastoid and the superior trapezius muscle; Tianzhu (BL10): Located 0.5 cun above the posterior hairline, 1.3 cun lateral to the midline, in the depression at the lateral margin of the trapezius muscle; Wangu (GB12): In the hollow just posteroinferior to the mastoid process; Lieque (LU7): Approximately 1.5 cun near the wrist crease, above the styloid process of the radius, in the recess between the tendons of the brachioradialis and abductor pollicis longus muscles; Zhaohai (KI6): Located in the depression below the medial malleolus; Jinjin (EX-HN12): On the left side of the lingual frenulum on the sublingual vein of the mouth; Yuye (EX-HN13): on the right side of the lingual frenulum of the oral cavity. Acupoint locations are described according to standard acupuncture textbooks, with minor modifications in wording for clarity (Artwork by Jing Luo and Qibin Yao).

Points: Baihui (GV20), Shenting (GV24), Yintang (EX-HN3), Fengchi (GB20), Wangu (GB12), Tianzhu (BL10), Lianquan (CV23), Zhaohai (KI6), Lieque (LU7); optional Yuye (EX-HN13), Jinjin (EX-HN12), and posterior pharyngeal wall needling. Acupoint location is shown in Figure 2.

Technique: Disposable needles with their needle sleeves will be used. GV20 subcutaneous at 15–30° (1 cun) with deqi; rotational manipulation 200 rpm for 2 min every 10 min; GV24 oblique toward GV20 (1 cun); EX-HN3 perpendicular 0.1–0.3 cun with reducing manipulation; GB20/GB12/BL10 oblique toward the thyroid cartilage; Needles will be retained for 20 min per session. The “deqi” sensation was defined as the patient experiencing soreness, numbness, heaviness, or distension at the needle site, which is commonly regarded as an indicator of effective needle stimulation in acupuncture practice.

Acupuncture at the EX-HN12/13 points and the posterior pharyngeal wall: The patient opens their mouth, the practitioner uses a sterile disposable acupuncture needle (0.30 × 75 mm) to perform superficial needling at the EX-HN12/13 points and the posterior pharyngeal wall. The needle is inserted shallowly into the mucosa and lightly tapped to induce mild stimulation and slight bleeding. The needle is not retained.

#### 2.5.2 SHAM-controlled group

Same point locations but non-penetrating sham needles with sleeves to avoid skin breach and deqi. Needles will be retained for 20 min per session. For EX-HN12/13 points and the posterior pharyngeal wall, blunt tips will lightly touch the mucosa without bleeding.

Schedule: Treatment will be administered once daily, 5 days per week, for 30 min per session over a total of 6 weeks, and will be delivered by licensed acupuncturists with more than 5 years of experience who have been trained in the study protocol.

#### 2.5.3 Healthy control group

A cohort of 20 healthy volunteers will undergo the identical rs-fMRI and DTI acquisition protocols. These baseline reference data will enable precise identification of PSD-specific alterations in brain functional networks and white-matter structure.

### 2.6 Concomitant therapy

All PSD patients will receive guideline-based pharmacotherapy and swallowing rehabilitation per institutional practice (15, 16).

Swallowing rehabilitation protocol: Indirect training will include lip-closure exercises, mandibular movement training, lingual mobility exercises, cold stimulation, articulation training, vocal cord adduction training, cough training, and stimulation to facilitate the swallowing reflex. Direct training will include positioning the patient in a seated posture or at a 30° reclined angle with the neck flexed forward. For patients with hemiplegia, elevate the affected side of the shoulder and back. Select appropriate foods based on the patient's specific condition and dietary habits. Start with small portions (1–4 ml) and gradually increase the amount. The feeding speed will be modified as needed, and techniques including dry swallowing, alternating swallowing, nodding swallowing, and lateral swallowing will be used to eliminate and swallow food remaining in both pyriform sinuses.

Swallowing training should be performed once daily for 30 min, five times weekly, for a total of 6 weeks of treatment.

To ensure consistency across participants, all therapists will follow a standardized rehabilitation procedure for dysphagia. Prior to trial initiation, therapists will receive unified training. Adherence to the rehabilitation protocol will be recorded in CRFs and monitored by the Data Monitoring Committee.

Throughout the entire trial, participants in both groups will not be permitted to receive any additional targeted therapies for dysphagia. If concomitant prescription medications are required, all relevant details will be documented in participants' case report forms (CRFs). These data will be independently reviewed by two blinded investigators.

### 2.7 Outcomes and assessment schedule

#### 2.7.1 Neuroimaging methods

MRI data acquisition: Rs-fMRI and DTI data will be acquired using a 3.0-T MRI system equipped with a 48-channel head coil (SIGNA Premier; GE Healthcare, Milwaukee, USA). Resting-state BOLD fMRI will use a two-dimensional gradient-echo echo-planar imaging (EPI) protocol, which has a multi-band (MB) factor of 2 and a parallel imaging factor of 2. The imaging parameters are as follows: repetition time (TR) = 2,000 ms, echo time (TE) = 30 ms, field of view (FOV) =224 mm, flip angle = 90°, matrix size = 64 × 64, in-plane voxel size = 3.5 × 3.5 mm^2^, slice thickness = 3.5 mm (no gap), comprising 43 axial slices with an interleaved acquisition order. Automated shimming and phase correction will be enabled. The total scan duration will be approximately 8 minutes, yielding 240 volumes. Subjects will be required to remain still with eyes closed, avoiding movement and falling asleep.

DTI acquisition: The DTI protocol will employ an axial spin-echo EPI sequence, b-value 1,000 s/mm^2^, comprising 35 non-collinear diffusion directions alongside one b0 image per repetition. The imaging parameters will be: TR = 5714 ms, TE = minimum (vendor-optimized), FOV = 224 mm, matrix size = 112 × 112, Isotropic voxel size = 2.0 × 2.0 × 2.0 mm^3^, and 76 axial slices covering the entire brain. The multiband factor will be 2, with fat saturation enabled, phase encoding in the right-left direction, and a number of excitations (NEX) of 2 for diffusion. Susceptibility and eddy-current correction will be executed during preprocessing.

Structural MRI: A 3D sagittal T1-weighted magnetization-prepared rapid acquisition gradient-echo (MPRAGE) will be taken for anatomic reference, segmentation, and cortical thickness and volume analyses, with parameters including: FOV = 256 mm, matrix size = 256 × 256, isotropic resolution = 1 mm^3^, 192 slices, flip angle = 8°, TE = 3.0 ms, TR = 7.3 ms, and inversion time (TI) = 1,321 ms. Furthermore, 3D sagittal T2 weighted and T2 fluid attenuated inversion recovery (FLAIR) (both 1 mm isotropic resolution) will be used for lesion delineation and quality control.

#### 2.7.2 Primary outcome

Derived imaging metrics will be calculated from the acquired rs-fMRI and DTI data as described below. The primary neuroimaging outcomes are defined as the changes from baseline to week 6 in prespecified rs-fMRI metrics (ALFF, ReHo, and FC) and DTI metrics (FA and MD). In addition, 20 healthy controls will undergo baseline rs-fMRI and DTI examinations to provide a reference for PSD-specific neuroimaging alterations.

Rs-fMRI: Rs-fMRI is extensively utilized to probe the neural mechanisms of PSD. The rs-fMRI markers include amplitude of low-frequency fluctuations (ALFF), regional homogeneity (ReHo), and functional connectivity (FC), which can be used to reflect brain function in patients with PSD. However, existing research primarily emphasizes activation in localized brain regions, with insufficient exploration of acupuncture's effect on functional networks in PSD patients (17).

Therefore, we employed rs-fMRI to analyze brain functional network connectivity, aiming to further clarify the central mechanisms underlying acupuncture treatment for PSD.

1. ALFF: ALFF reflects the local activity level of specific brain regions by calculating signal strength in the 0.01–0.1 Hz frequency band. It is commonly utilized to explore activation patterns in various brain areas, facilitating our understanding of functional changes across different regions following a stroke, particularly those related to swallowing (18).
2. ReHo: ReHo can capture local coherence changes within swallowing-network nodes (e.g., insula, M1, SMA) in PSD by measuring the temporal synchrony between a given brain region and its adjacent voxels, thereby assessing the consistency of neuronal activity within that region (19).
3. FC: Functional connectivity assesses the interconnections between brain regions based on correlations in temporal sequences. Seed-based analysis (e.g., bilateral insula, M1 orofacial area, SMA) and independent component analysis (ICA) employ data-driven methods to capture broader network-level modulation. Evaluating overall connectivity patterns enhances our understanding of how acupuncture may improve neural network function in PSD patients (20, 21).

DTI: DTI facilitates the reconstruction, visualization, and quantification of structural and fiber connectivity within the brain (22). Fractional anisotropy (FA) serves as an index of the integrity of white matter microstructure, while mean diffusivity (MD) is indicative of pathology, including edema and necrosis (23). Consequently, DTI provides essential insights into structural remodeling and fiber tract reorganization in patients with PSD (24).

By integrating rs-fMRI and DTI through multimodal analysis, both functional and structural connectivity within the brain can be simultaneously examined. This approach provides more comprehensive information on neural networks and may help elucidate the structural-functional coupling mechanisms underlying acupuncture-mediated recovery of swallowing function. This contributes to a deeper understanding of the multidimensional mechanisms underlying acupuncture-induced neural reorganization in patients with PSD.

#### 2.7.3 Secondary outcomes: mean changes in SSA, VFSS-based measures (MBSImP and PAS), MoCA, and SWAL-QOL scores during the study period

(1) SSA (Standardized Swallowing Assessment): The SSA consists of three components: clinical examination, 5 ml and 60 ml water swallowing tests (25). Higher scores reflect more severe swallowing dysfunction. Existing evidence suggests that the SSA offers good sensitivity and specificity when used to assess PSD patients (26).

(2) VFSS (Video fluoroscopic Swallowing Study)-based measures: VFSS is considered one of the most accurate methods for the diagnosis of dysphagia and is conducted using X-ray fluoroscopy to evaluate the transit of food boluses with contrast agent from the oral cavity to the esophagus in both anteroposterior and lateral views (27). Swallowing impairment will be quantified using the Modified Barium Swallow Impairment Profile (MBSImP), which scores standardized physiologic components observed on VFSS across the oral and pharyngeal phases, including pharyngoesophageal segment opening, and also incorporates upright esophageal clearance as a screening component, using ordinal impairment ratings (0 = normal; higher scores = more severe impairment), thereby generating a profile-based characterization of swallowing dysfunction (28). Because airway invasion is a critical safety dimension, penetration/aspiration events during VFSS will additionally be graded using the Penetration–Aspiration Scale (PAS), an 8-point ordinal scale in which PAS = 1 indicates no airway entry and higher scores indicate increasing penetration/aspiration severity (29). MBSImP (with PAS as a complementary safety indicator), together with SSA, will enable a comprehensive evaluation of swallowing function and therapeutic response in PSD.

(3) MoCA (Montreal Cognitive Assessment): In traditional Chinese medicine, “Spirit” refers to the sum of vital activities and mental processes, including cognition, perception, and emotion. Cognitive function is therefore considered part of “Spirit” and reflects its regulatory capacity, which also influences cerebral function (30). Patients with PSD frequently present with concomitant cognitive impairment (31). The MoCA serves as a widely adopted tool for cognitive evaluation with strong reliability and validity (32). It assesses eight domains: attention, orientation, fluency, naming, visuospatial skills, abstraction, delayed recall, and sentence repetition. The total score is 30, with one additional point added for individuals with fewer than 12 years of education. A lower score indicates greater cognitive impairment. It will be used to quantify changes in cognitive function attributable to acupuncture.

(4) SWAL-QOL (Swallowing Quality-of-Life Questionnaire): The SWAL-QOL comprises 44 items covering appetite, mealtime duration, food selection, sleep, and psychological status, and is specifically developed to measure the quality of life of individuals with PSD (33). A higher score corresponds to better quality of life. It will be employed to evaluate the impact of acupuncture on patients' quality of life.

Assessment schedule: baseline (week 0), weeks 2 and 4, and week 6 (end-of-treatment). All assessments will be performed by trained, blinded evaluators.

### 2.8 Quality control (QC) & exclusion rules

Head Motion (rs-fMRI): Frames exhibiting a framewise displacement (FD) greater than 0.5 mm will be scrubbed; datasets with an average FD > 0.2 mm or containing more than 20% high-motion frames will be flagged for exclusion or sensitivity analyses. Summary statistics and outlier count for FD and DVARS will be reported (34, 35).

Image Integrity: Visual quality control will assess co-registration and normalization accuracy, field-of-view coverage, and the presence of artifacts (e.g., ghosting, spikes) (36).

DTI Artifacts: Automatic detection and removal of corrupted directions or slices will be implemented, alongside a review for residual distortion post-TOPUP and EDDY correction (37).

Reproducibility: All parameters, masks, and scripts will be version-controlled (38).

### 2.9 Sample size

This study is designed as an exploratory, mechanism-oriented neuroimaging trial. At present, there are limited prior data from sham-controlled acupuncture studies in post-stroke dysphagia that combine multimodal MRI outcomes with comparable clinical endpoints, which makes a formal power calculation based on a single anticipated effect size difficult. Therefore, the planned sample size was determined with reference to previous neuroimaging studies, practical feasibility, and the exploratory nature of the present protocol. Given the characteristics and high costs associated with neuroimaging studies, sample sizes are generally smaller compared with conventional clinical trials, and there is currently no standardized consensus regarding the minimum sample size required to achieve stable statistical power. A study in 2002 reported that a sample size of 12 subjects was sufficient to provide relatively stable statistical power in rs-fMRI research (39). In 2007, another study using auditory stimulation of rs-fMRI suggested that a sample size of 13–15 subjects was adequate for stable statistical inference (40). In 2011, investigators compared sample sizes of 5, 8, 14, 17, and 21 subjects under otherwise consistent conditions. They observed that smaller sample sizes (e.g., 5 or 8) produced fewer activation regions with greater variability, whereas larger sample sizes yielded more activation clusters and reduced intergroup differences (41). Ultimately, the literature recommends that, where ethically and practically feasible, neuroimaging trials should include at least 20 subjects per group (42).

Taking into account the constraints of study duration, patient hospitalization time, and available manpower, and with reference to previous clinical research, we determined that a randomized controlled trial with 20 participants per group (40 in total) would be appropriate. Considering an expected 15% dropout rate, the final planned sample size is 46 participants [40 × (1 + 15%) = 46].

### 2.10 Data collection

Data collection will be performed by therapists employing standardized CRFs. Two independent researchers will then enter the data into a secure database. All analyses will be conducted under the supervision of an independent data monitor to ensure accuracy, consistency, and data integrity. For participants who withdraw, their number and reasons (particularly those related to AEs) will be carefully documented.

### 2.11 Statistical analysis

#### 2.11.1 fMRI data analysis

Preprocessing and analyses will be conducted with SPM12, DPABI, and FSL. Functional and structural connectivity will be analyzed using independent component analysis (ICA) and canonical correlation analysis (CCA) (43–45). ROI-based analyses will prioritize prespecified swallowing-related hubs (the insula, basal ganglia, frontal cortex, M1, SMA, corona radiata, and S1) as the primary ROI set for functional and structural connectivity tests. Network-level extensions (e.g., the default mode network (DMN), the frontal-temporal-sensory-motor circuit, the sensory-motor-insular-thalamic circuit and the insula-cerebellum-occipital circuit) will be reported as secondary/supportive analyses with appropriate multiple-comparison control. Structure–function coupling will be assessed by examining the relationship between functional connectivity and structural connectivity between corresponding brain regions. Functional connectivity (FC) will be calculated as the Pearson correlation between the BOLD time series of predefined regions of interest (ROIs). Structural connectivity will be derived from diffusion tensor imaging using probabilistic tractography to identify white-matter fiber tracts connecting the same ROI pairs. For each tract, the mean fractional anisotropy (FA) along the reconstructed fibers will be calculated to represent the integrity of the structural pathway. Structure–function coupling will then be evaluated by assessing the relationship between FC strength and tract-specific FA values across ROI pairs (46). In addition to the hypothesis-driven ROI analyses, exploratory whole-brain analyses will be conducted to identify potential PSD-related brain regions beyond the predefined swallowing network. Voxel-wise comparisons of rs-fMRI metrics (e.g., ALFF and ReHo) will be performed across the whole brain between groups. Statistical analyses will be conducted using a general linear model framework, and multiple comparisons will be controlled using false discovery rate (FDR) correction (p < 0.05). This will allow us to assess how acupuncture modulates the interaction between structural integrity and functional network organization in PSD.

To correct for multiple comparisons, we will apply the false discovery rate (FDR) at the voxel level and Bonferroni correction for network-level metric (47).

Statistical Modeling: For clinical outcomes and imaging metrics, linear mixed-effects models will be employed to assess within-subject change (baseline vs. week 6) and between-group differences (acupuncture vs. control), while adjusting for sex, age, stroke severity, and lesion volume (48). Correlation analyses will test associations between rs-fMRI/DTI metrics and clinical improvement in swallowing function (SSA/VFSS-based measures/MoCA/SWAL-QOL scales) (49, 50). This will aid in assessing whether alterations in brain networks correlate with improvements in swallowing function, thereby enhancing the clinical interpretability of imaging findings. Depending on the assumptions of normality and linearity, analyses will use either Pearson's correlation coefficient or Spearman's rank correlation coefficient.

#### 2.11.2 Clinical data analysis

All randomized participants will be included in the preliminary analysis according to the intention-to-treat (ITT) principle. Data will be analyzed with SPSS v25.0 (IBM, Armonk, NY, USA). A two-tailed *p*-value of ≤ 0.05 will be taken to indicate statistical significance, and continuous data will be reported as mean ± standard deviation (SD). For normally distributed continuous variables, an independent-samples *t*-test will be used, whereas for non-normally distributed data, the Mann–Whitney *U* test will be applied. Categorical and ordinal variables will be expressed as percentages (%) and analyzed using the chi-square test when expected cell counts are adequate (typically all expected counts ≥5), or Fisher's exact test when expected counts are small. Ordinal data will be analyzed using the rank-sum test.

The incidence of adverse events will be summarized in the safety analysis and compared between groups using the chi-square test or Fisher's exact test, as appropriate. Missing data for participants who withdraw or deviate from the protocol will be handled using listwise deletion or multiple imputation methods.

fMRI data will be analyzed by experienced imaging specialists. All statistical analyses will be performed by independent data analysts who are blinded to group allocation, thereby minimizing potential bias.

### 2.12 Adverse events and safety monitoring

All adverse events (AEs) will be recorded in the case report form (CRF) and managed within 24 hours. A study physician will evaluate each AE and document AE type, onset/duration, management, outcome, CTCAE grade (1–5), and causality (definitely/probably/possibly/unlikely/unrelated).

AEs will be graded according to the Common Terminology Criteria for Adverse Events (CTCAE): Grade 1 (mild), Grade 2 (moderate), Grade 3 (severe), Grade 4 (life-threatening), and Grade 5 (death). Serious adverse events (SAEs) (fatal, life-threatening, requiring/prolonging hospitalization, or causing persistent/significant disability) and emergencies will be reported to the ethics committee and relevant institutional personnel within 24 hours of awareness.

Participants may withdraw at any time; investigators may discontinue the intervention if continuation is deemed unsafe. Participants experiencing AEs will be followed until stabilization/resolution. The principal investigator will review AE reports regularly, and a safety review meeting will be convened if necessary. Potential acupuncture-related AEs include syncope, needle retention/breakage, local hematoma, and localized infection; participants with severe intervention-related AEs will be withdrawn immediately.

### 2.13 SPIRIT compliance

**Figure 3:**
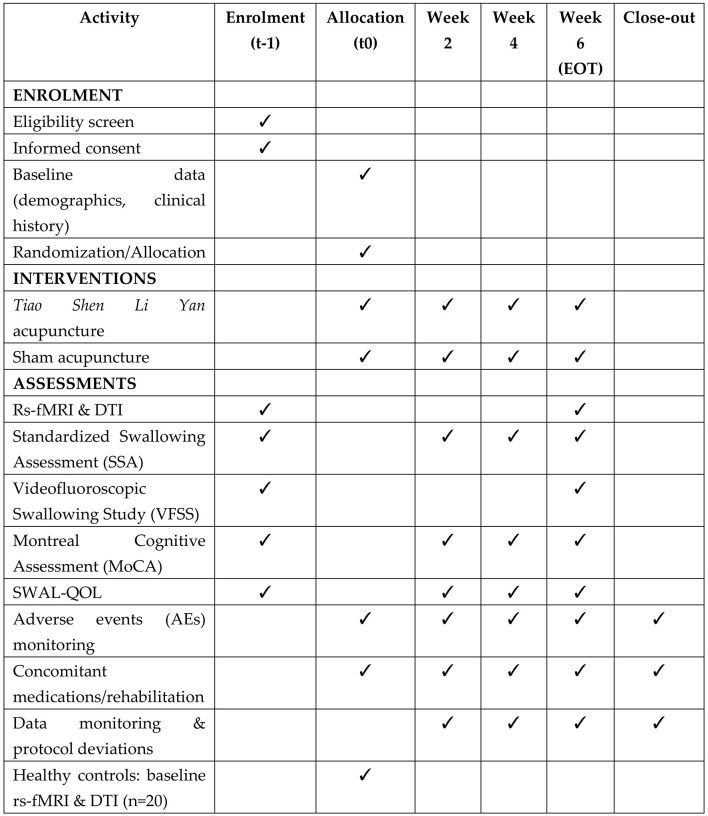
SPIRIT schedule of enrolment, interventions, and assessments. EOT, End of Treatment; rs-fMRI/DTI at baseline (t0), and Week 6; SSA at t0/Weeks 2/4/6; VFSS/MoCA/SWAL-QOL at t0 and Week 6; AEs monitored throughout.

This protocol adheres to SPIRIT 2013; the completed SPIRIT checklist and the SPIRIT figure are provided in the Supplementary material. Figure 3 summarizes the enrolment, intervention and evaluation calendar (SPIRIT).

### 2.14 Data monitoring committee (DMC)

DMC consists of experienced biostatisticians from Guangzhou University of Traditional Chinese Medicine. They do not participate in daily research or data processing of the study. The committee is responsible for periodically reviewing trial conduct, recruitment, data completeness and quality, protocol adherence (including adherence to allocation concealment procedures), adverse events, the safeguarding of participant privacy, and overall participant safety. Based on these reviews, the DMC may provide independent recommendations to the principal investigator and the Medical Ethics Committee on whether the trial should continue as planned, be modified, or be terminated early. The DMC operates independently of the investigators and has no competing interests related to this study.

### 2.15 Trial status

The trial is now in its recruitment period, and the first subject was enrolled on 23 July 2024. Recruitment is planned to continue until 31 December 2025, and each participant will be followed for 6 weeks after enrolment. The current protocol version is 1.1 (dated 17 October 2025). At the time of manuscript submission, patient enrolment and follow-up are ongoing; the data remain blinded and no primary outcome analyses have been conducted.

## 3 Discussion

Currently, acupuncture has demonstrated significant efficacy in the treatment of PSD and is recommended in clinical guidelines (16); however, its central mechanisms remain unclear. Previous fMRI-related studies have primarily focused on the activation effects of acupuncture on PSD brain regions, whilst in-depth exploration of the mechanisms underlying functional network reorganization remains relatively limited (51). This study employs multimodal rs-fMRI and DTI to investigate the mechanisms of brain functional network reorganization in patients with PSD treated with Tiao Shen Li Yan acupuncture. To our knowledge, this is the first study to elucidate changes in acupuncture-induced brain functional connectivity patterns and structural alterations in inter-network white-matter pathways, thereby exploring the underlying structure-function coupling mechanisms, while also using healthy individuals as imaging baseline controls to define PSD-specific network alterations. Through objective neuroimaging evidence and clinical data, this study aims to provide crucial support for advancing acupuncture research and clinical practice.

The Tiao Shen Li Yan acupuncture is grounded in traditional Chinese medical (TCM) pathophysiology, centered on the notion that dysregulation of the “Yuan-Shen” (vital spirit) disrupts the guidance of qi and thereby impairs pharyngeal function. Anchored in the TCM holistic view that the brain is the residence of the Yuan-Shen and governs the whole body, the therapy operates on two levels. At the systemic level, modulation of the Yuan-Shen is intended to restore normal central control of swallowing. At the local level, “facilitating the pharyngeal orifices” aims to improve swallowing function; in addition, needling of throat-related acupoints provides afferent feedback that may promote reorganization within the central swallowing circuitry. This synergy between systemic and local interventions is designed to comprehensively recalibrate swallowing function and thereby enhance therapeutic efficacy. Experimental evidence suggests that electroacupuncture at Lianquan (CV23) can activate excitatory neurons in the primary motor cortex and modulate swallowing-related musculature via the M1-parabrachial nucleus-nucleus tractus solitarius pathway, improving swallowing function (52). Furthermore, acupuncture at Fengchi (GB20) has been reported to ameliorate dysphagia by modulating cerebral blood flow and neural function (53). The acupoints Baihui (GV20), Shenting (GV24), and Yintang (EX-HN3)—key nodes for regulating “shen” in stroke rehabilitation—have been associated with anti-inflammatory effects, network-level neuromodulation, and improved cerebral circulation (54). Patients with PSD often present with varying degrees of cognitive impairment (31), which in traditional Chinese medicine may be interpreted as a disturbance of “Shen.” Accordingly, the MoCA will be used as a clinical outcome measure to evaluate changes in cognitive function. In combination, these acupoints provide a multi-target therapeutic strategy for PSD, collectively achieving the intended effect of “regulating shen and facilitating the throat.” Although our preliminary clinical work has demonstrated the clinical efficacy of the Tiao Shen Li Yan acupuncture approach in PSD, its central mechanisms remain to be elucidated (8).

Post-stroke dysphagia (PSD) occurs when cerebral infarction disrupts the distributed neural system that supports swallowing, leading to abnormalities in both network connectivity and white-matter integrity. Importantly, converging neuroimaging evidence suggests that PSD is best conceptualized as a network-level disorder rather than a focal lesion effect, involving coordinated dysfunction across cortical and subcortical hubs. At the cortical level, swallowing-related control engages the sensorimotor cortex (M1/S1/S2), SMA, and frontal regions (including IFG), and interacts closely with subcortical structures such as the thalamus, insula, and basal ganglia, as well as bottleneck white-matter regions (e.g., corona radiata/internal capsule) (55). Recent work has further described modular organization within the swallowing connectome (e.g., insula, cerebellum, sensorimotor–cingulate, IFG–S2–corpus callosum–basal ganglia–thalamus, and premotor–posterior parietal modules), which can be arranged into higher-order circuits supporting swallowing control and compensation (55). Notably, prior rs-fMRI studies in PSD have reported both regional activity alterations and connectivity abnormalities across these hubs, including the sensorimotor cortex, insula, cerebellum, cingulate, thalamus, and basal ganglia (56). Across studies, the most frequently implicated loci include the insula, basal ganglia, frontal cortex, M1, SMA, corona radiata, and S1(57). However, brain network–level evidence remains limited, and existing studies have only partially examined how these regions reorganize as integrated circuits during recovery. For example, rs-fMRI and fNIRS studies suggest abnormalities in DMN and reduced connectivity between the frontal–temporal–sensory–motor circuit and the sensory–motor–insular–thalamic circuit; moreover, altered insula–cerebellum–occipital functional connectivity has been associated with dysphagia severity (51, 58).

Therefore, in the present study we explicitly separate primary and secondary analyses. The primary (confirmatory) analyses anchor structure-function coupling tests to a prespecified ROI set that captures core swallowing-related hubs with consistent PSD abnormalities (insula, basal ganglia, frontal cortex, M1, SMA, corona radiata, and S1). The secondary (supportive) analyses extend to larger-scale circuits (DMN, frontal–temporal–sensory–motor, sensory–motor–insular–thalamic, and insula–cerebellum–occipital circuits) and are interpreted as hypothesis-generating.

DTI provides complementary insight into the microstructural substrates of PSD. The severity of white-matter injury—reflected by FA and MD metrics—is closely related to the severity of dysphagia, and adaptive white-matter changes may accompany recovery (59). Stroke is associated with disturbances of both functional and structural networks and with state-dependent reductions in functional-structural coupling (60). Therefore, this study included 20 healthy controls as a baseline reference to identify network sites with PSD abnormalities. Combined rs-fMRI and DTI analyses of SC-FC coupling sensitively index clinical impairment and recovery after PSD (46, 60).

Rs-fMRI studies indicate that acupuncture can activate swallowing-related cortical regions in PSD and enhance activity within central swallowing hubs, thereby promoting functional recovery of the cortical swallowing centers. FNIRS evidence suggests that acupuncture facilitates frontotemporal-sensorimotor network integration (61). However, PSD involves not only widespread cortico-cortical injury but also disruption of cortico-subcortical networks, and the mechanisms of structure-function reorganization remain insufficiently understood (51).

Building on this analysis hierarchy, we will examine whether acupuncture-related changes in structure-function coupling and circuit-level functional connectivity track clinical improvement over the 6-week intervention period, using healthy controls as a baseline reference.

We hypothesize that the Tiao Shen Li Yan acupuncture may improve swallowing function in PSD by enhancing integration within swallowing-related functional networks and promoting microstructural repair of inter-network white-matter pathways, thereby strengthening structure-function coupling across swallowing networks and facilitating functional reorganization of brain networks. In addition to the predefined ROI-based analyses, exploratory whole-brain analyses will also be performed to identify potential PSD-related brain regions that may not be included in the current swallowing network framework. These regions could become targets for future investigations. Importantly, structural and functional connectivity do not necessarily change in parallel during post-stroke recovery. Improvements in functional connectivity in the absence of structural recovery may reflect compensatory reorganization rather than true restoration (62), whereas structural recovery without corresponding functional gains may indicate suboptimal recovery. Therefore, quantifying structure–function coupling may provide mechanistic insight beyond assessing functional or structural metrics alone, by characterizing whether and how functional network reorganization remains constrained by (or diverges from) the underlying structural scaffold.

Overall, a major strength of this study is that it combines rs-fMRI and DTI to establish an “imaging-to-clinic” evidence chain that closely links clinical efficacy with mechanistic evidence. This approach is intended to delineate the structure-function coupling mechanisms by which the Tiao Shen Li Yan acupuncture modulates swallowing networks in PSD, and reveal the mechanism of brain functional network reorganization in PSD patients through acupuncture, while the inclusion of healthy controls enhances clinical relevance and interpretability.

However, this study has several limitations. For instance, the sample size is small and there is no sustained long-term follow-up. Because of budget and time constraints, only 46 patients will be included in this randomized controlled trial with a 6-week observation period. Future research should conduct multicentre trials, expand sample sizes, and extend follow-up periods to 3 months or longer to assess the persistence of clinical efficacy and neuroimaging effects, thereby enabling more in-depth exploration. Secondly, due to the nature of acupuncture research, complete blinding of the intervention is not feasible, which may introduce a risk of bias. Nevertheless, implementing random allocation concealment and maintaining complete blinding of evaluators and statisticians will help mitigate potential bias, thereby ensuring the rigor and credibility of the trial. In addition, task-based fMRI during swallowing was not included in the present protocol. Although task fMRI may provide more direct information on swallowing-related motor control and enable effective connectivity analysis, performing swallowing tasks during MRI scanning may introduce substantial motion-related artifacts and technical challenges, particularly in patients with PSD. Future studies could consider incorporating optimized task paradigms or multimodal approaches to further investigate task-specific neural mechanisms.

## 4 Conclusions

In conclusion, this randomized controlled trial employing rs-fMRI and DTI is designed to assess both the clinical efficacy and the central neural mechanisms of Tiao Shen Li Yan acupuncture in PSD. The results are expected to provide objective evidence regarding the clinical efficacy and neural mechanisms of acupuncture and to serve as a valuable reference for evidence-based PSD management guidelines.
