# Supplementary material for: Multimodal neuroimaging protocol to explore the neural mechanisms of Tiao Shen Li Yan acupuncture in post-stroke dysphagia: a randomized sham-controlled clinical trial
Source: Front Neurol. 2026 Jun 19;17:1764500. doi: 10.3389/fneur.2026.1764500 (PMC13333810; doi:10.3389/fneur.2026.1764500)
Supplement: Supplementary file 1 [file Supplementary_file_1.zip › supplementary/Completed SPIRIT 2013 Checklist.docx]

**Table S1. Completed SPIRIT 2013 Checklist**

Title of study:

Multimodal Neuroimaging Protocol to Explore the Neural Mechanisms of Tiao Shen Li Yan Acupuncture in Post-Stroke Dysphagia: A Randomized Sham-Controlled Clinical Trial

Journal:

Frontiers in Neurology – Neurorehabilitation

Manuscript type:

Study Protocol

Trial registration:

Chinese Clinical Trial Registry (ChiCTR2400086748);

Registered 10 July 2024; last updated 30 October 2025.

Protocol version and date:

Protocol Version 1.1, dated 17 October 2025.

Trial sponsor / funders:

Shenzhen Futian District Health Bureau (FTWS057);

Sanming Project of Medicine in Shenzhen (SZSM201502044);

Shenzhen Chinese Medicine Key Specialty Construction Project (No. ZYTS019).

Principal Investigator:

Jing Luo, MD

Shenzhen Hospital (Futian) of Guangzhou University of Chinese Medicine, Shenzhen, China.

Corresponding authors:

Prof. Zhenhua Xu

E-mail: xzh197011@163.com

Prof. Zhentao Zuo

E-mail: zuozt@ibp.ac.cn

### Administrative information

| **Section** | **Item** | **How addressed in this protocol** | **Location in this protocol** |
| --- | --- | --- | --- |
| Administrative information | Title | Descriptive title identifies design (randomized, single-blind, sham-controlled), population (PSD), intervention (Tiao Shen Li Yan acupuncture), and multimodal neuroimaging (rs-fMRI and DTI). | Title page; Abstract; Section 1. Introduction |
| Administrative information | Trial registration | Registry name (Chinese Clinical Trial Registry), registration number (ChiCTR2400086748), prospective registration date and update date reported. | Abstract (Trial Registration); Section 2.1 Registration and Trial Design; Ethics and Dissemination |
| Administrative information | Protocol version | Current protocol version and date are specified; amendment clarifies outcome hierarchy without changing sample size, time points, risk profile, or statistical plan. | Section “Data Monitoring Committee” and “ Trial Status”; Ethics and Dissemination |
| Administrative information | Funding | Names of funding bodies and grant/ project numbers are listed. | Funding section |
| Administrative information | Roles and responsibilities | Identifies principal investigator, corresponding authors, and contributors; details roles by CRediT taxonomy (conceptualization, methodology, imaging, statistics, supervision, etc.). | Author list; Author Contributions section |

### Introduction

| **Section** | **Item** | **How addressed in this protocol** | **Location in this protocol** |
| --- | --- | --- | --- |
| Introduction | Background and rationale | Summarizes epidemiology and clinical impact of PSD; limitations of current rehabilitation and pharmacologic treatments; TCM theory of “Tiao Shen Li Yan”; previous acupuncture and neuroimaging studies; remaining gaps in understanding brain network mechanisms. | Section 1. Introduction; early Discussion paragraphs |
| Introduction | Choice of comparator | Justifies sham acupuncture at same acupoints using non-penetrating needles without deqi as control for non-specific and expectancy effects, while maintaining patient blinding as far as possible. | Section 2.5 Interventions – Sham-controlled group |
| Introduction | Objectives / hypotheses | States primary objective: to evaluate clinical efficacy and central neural mechanisms (functional and structural connectivity and structure–function coupling) of Tiao Shen Li Yan acupuncture in PSD; hypothesizes enhancement of swallowing-related brain networks and white-matter microstructure leading to improved dysphagia. | Section 1. Introduction ; Section 2.7.1 Primary outcome; Discussion |
| Introduction | Trial design | Describes the trial as a single-center, randomized, single-blind, sham-controlled, parallel-group clinical trial with 1:1 allocation and an additional healthy control imaging cohort. | Abstract (Methods); Section 2.1 Registration and Trial Design |

### Methods: Participants, interventions, and outcomes

| **Section** | **Item** | **How addressed in this protocol** | **Location in this protocol** |
| --- | --- | --- | --- |
| Methods: Participants, interventions, and outcomes | Study setting | Specifies a single tertiary teaching TCM hospital in Shenzhen (Shenzhen Hospital (Futian) of Guangzhou University of Chinese Medicine); recruitment from inpatient and outpatient departments; neuroimaging acquired on site. | Section 2.2 Study Setting and Recruitment |
| Methods: Participants, interventions, and outcomes | Eligibility criteria – PSD patients | Defines inclusion criteria (diagnosed ischemic stroke with imaging confirmation, dysphagia per guidelines, age 30–80, onset 2 weeks–6 months, stable vital signs, clear consciousness, able to cooperate, signed informed consent); and exclusion criteria (coma, Parkinson’s disease, dementia, severe organ failure, no cerebrovascular lesion on imaging, poor adherence, major psychiatric disease, MRI contraindications). | Section 2.3 Eligibility Criteria |
| Methods: Participants, interventions, and outcomes | Eligibility criteria – healthy controls | Healthy volunteers aged 30–80 with normal swallowing, no neurological disease, no history of stroke or pharyngeal surgery/tumor; normal WST; able to undergo MRI; provide baseline rs-fMRI and DTI only. | Section 2.5 Interventions – Healthy Control Group; Section 2.7.1 Primary outcome |
| Methods: Participants, interventions, and outcomes | Interventions – acupuncture group | Details Tiao Shen Li Yan acupuncture prescription: acupoint locations (GV20, GV24, EX-HN3, CV23, GB20, BL10, GB12, LU7, KI6, optional EX-HN12, EX-HN13 and posterior pharyngeal wall), needle type, insertion depth and angle, manipulation, elicitation of deqi, retention time (~30 min), treatment frequency (5 times/week) and duration (6 weeks). | Section 2.5 Interventions – Tiao Shen Li Yan acupuncture |
| Methods: Participants, interventions, and outcomes | Interventions – sham control | Describes sham acupuncture using non-penetrating needles at the same acupoints without skin penetration or deqi, same session duration and frequency, mimicking procedure while minimizing specific acupuncture effects. | Section 2.5 Interventions – Sham-controlled group |
| Methods: Participants, interventions, and outcomes | Concomitant care | States that all PSD patients receive guideline-based pharmacotherapy and standardized swallowing rehabilitation; additional dysphagia-targeted therapies outside the protocol are not permitted; concomitant medications and rehabilitation are recorded. | Section 2.6 Concomitant therapy; Swallowing rehabilitation protocol |
| Methods: Participants, interventions, and outcomes | Criteria for discontinuation / modification | Participants may withdraw at any time; investigators may discontinue acupuncture for severe or intolerable adverse events or medical reasons; SAEs and unexpected events prompt review by ethics/DMC and may lead to modification or termination recommendations. | Section 2.13 Adverse Events and Safety Monitoring |
| Methods: Participants, interventions, and outcomes | Strategies to improve adherence | Uses standardized acupuncture protocol delivered by trained licensed acupuncturists; fixed treatment schedule (5×/week for 6 weeks); attendance, protocol deviations, and reasons for withdrawal recorded in CRFs; data monitored by independent staff. | Section 2.5 Interventions; Section 2.11 Data collection |
| Methods: Participants, interventions, and outcomes | Primary outcome | Primary outcome is change from baseline to Week 6 in prespecified rs-fMRI metrics (ALFF, ReHo, FC) and DTI metrics (FA, MD) in PSD patients; healthy controls provide reference baseline neuroimaging data for identifying PSD-specific alterations. | Section 2.7.1 Primary outcome; Section 2.8 Neuroimaging Methods; Section 2.12 Statistical Analysis |
| Methods: Participants, interventions, and outcomes | Secondary outcomes | Secondary outcomes are changes in Standardized Swallowing Assessment (SSA), videofluoroscopic swallowing study (VFSS) scores, Montreal Cognitive Assessment (MoCA), and Swallowing Quality-of-Life Questionnaire (SWAL-QOL) scores across baseline, Week 2, Week 4, and Week 6. Each instrument’s domains, scoring, and interpretation are described. | Section 2.7.2 Secondary outcomes (SSA, VFSS, MoCA, SWAL-QOL); Table 1 |
| Methods: Participants, interventions, and outcomes | Participant timeline | Provides a schedule of enrolment, randomization, interventions, neuroimaging (baseline and Week 6), and clinical assessments at baseline, Week 2, Week 4, and Week 6; summarized in a SPIRIT-style time–event table and figure. | Section 2.7 Outcomes and Assessment Schedule; Table 1; SPIRIT Figure |
| Methods: Participants, interventions, and outcomes | Sample size justification | Plans to enroll 46 PSD patients (23 per group) based on previous neuroimaging power considerations and feasibility, targeting at least 20 per group and accounting for ~15% attrition; plus 20 healthy controls for imaging reference. | Section 2.10 Sample Size |
| Methods: Participants, interventions, and outcomes | Recruitment strategy | Recruitment through inpatient and outpatient clinics using notices and clinician referrals; feasibility supported by routine PSD caseload; recruitment and trial status summarized. | Section 2.2 Study Setting and Recruitment; Section “Trial Status” |

### Methods: Assignment of interventions (allocation)

| **Section** | **Item** | **How addressed in this protocol** | **Location in this protocol** |
| --- | --- | --- | --- |
| Methods: Assignment of interventions | Sequence generation | Uses simple randomization with a random number table to allocate PSD patients 1:1 to acupuncture vs sham acupuncture. | Section 2.4 Randomization and Blinding |
| Methods: Assignment of interventions | Allocation concealment | Allocation sequence concealed using opaque, sequentially numbered, sealed envelopes stored securely; envelopes opened sequentially only after participant enrolment. | Section 2.4 Randomization and Blinding |
| Methods: Assignment of interventions | Implementation | An independent researcher prepares the randomization list and envelopes; the study coordinator enrolls eligible participants and assigns ID numbers; acupuncturists open the envelopes and deliver the assigned intervention. | Section 2.4 Randomization and Blinding |
| Methods: Assignment of interventions | Blinding | Participants, clinical outcome assessors, imaging analysts, data managers, and statisticians are blinded to group allocation; acupuncturists can not be fully blinded due to the nature of acupuncture; healthy controls know they are not randomized. | Section 2.4 Randomization and Blinding |
| Methods: Assignment of interventions | Emergency unblinding | Unblinding will occur only after database lock and completion of primary analyses, unless earlier unblinding is required for safety or ethics reasons; any such unblinding is overseen by the ethics committee and DMC. | Section 2.4 Randomization and Blinding; Section 2.13 Adverse Events and Safety Monitoring; Section 2.15 Data Monitoring Committee and Trial Status |

### Methods: Data collection, management, and analysis

| **Section** | **Item** | **How addressed in this protocol** | **Location in this protocol** |
| --- | --- | --- | --- |
| Methods: Data collection, management, and analysis | Outcome data collection | Uses standardized Chinese versions of SSA, VFSS, MoCA, and SWAL-QOL; assessments performed by trained, blinded evaluators according to published procedures; neuroimaging data acquired with detailed MRI parameters and consistent protocols. | Section 2.7 Outcomes and Assessment Schedule; Section 2.8 Neuroimaging Methods |
| Methods: Data collection, management, and analysis | Baseline and other data | Baseline demographics, stroke characteristics (lesion location, NIHSS or similar), comorbidities, and concomitant treatments collected at enrolment; all data recorded in CRFs and entered into a secure database. | Section 2.2 Study Setting and Recruitment; Section 2.7 Outcomes; Section 2.11 Data collection |
| Methods: Data collection, management, and analysis | Data management and quality assurance | Data recorded on standardized CRFs; double data entry by two independent researchers; independent monitor checks for completeness and consistency; imaging quality control includes head-motion thresholds, visual inspection, and automated DTI artifact detection; all scripts and parameters version-controlled. | Section 2.9 Quality Control & Exclusion Rules; Section 2.11 Data collection; Section 2.12 Statistical Analysis– fMRI data analysis |
| Methods: Data collection, management, and analysis | Statistical methods – imaging | fMRI preprocessing with SPM12/DPABI/FSL; ALFF, ReHo and FC analyses; multimodal integration using joint ICA and canonical correlation analysis; structure–function coupling assessed by correlations between FC strength and FA/MD of corresponding tracts; linear mixed-effects models used to evaluate within-subject changes and between-group differences. | Section 2.12 Statistical Analysis – fMRI data analysis; Statistical Modeling |
| Methods: Data collection, management, and analysis | Statistical methods – clinical outcomes | Clinical outcomes analyzed under ITT principle; trajectories of SSA, VFSS, MoCA, and SWAL-QOL modeled by linear mixed-effects models; continuous variables summarized as mean ± SD; t-tests for normally distributed data, Mann–Whitney U for non-normal data; categorical/ordinal variables compared by chi-square or Fisher’s exact test; ordinal data also analyzed by rank-sum tests. | Section 2.12 Statistical Analysis – Clinical data analysis |
| Methods: Data collection, management, and analysis | Missing data | For participants who withdraw or deviate from protocol, missing data will be handled using listwise deletion or multiple imputation, depending on missingness patterns and assumptions. | Section 2.12 Statistical Analysis – Clinical data analysis |
| Methods: Data collection, management, and analysis | Multiplicity and multiple comparisons | Multiple comparison correction for imaging analyses will use FDR at the voxel level and Bonferroni correction at the network-metric level; no formal interim efficacy analysis is planned. | Section 2.12 Statistical Analysis –fMRI data analysis; Section 2.15 Data Monitoring Committee and Trial Status |

### Methods: Monitoring

| **Section** | **Item** | **How addressed in this protocol** | **Location in this protocol** |
| --- | --- | --- | --- |
| Methods: Monitoring | Data Monitoring Committee | Describes an independent DMC composed of experienced biostatisticians from Guangzhou University of Chinese Medicine who are not involved in daily trial conduct or primary analysis; DMC reviews trial conduct, recruitment, data quality, protocol adherence, adverse events, and participant safety, and can recommend modification or termination. | Section 2.15 Data Monitoring Committee and Trial Status |
| Methods: Monitoring | Interim analyses and stopping guidelines | No formal interim efficacy analysis planned; DMC and ethics committee periodically review safety and trial progress; serious or unexpected events may prompt recommendations to modify or stop the trial. | Section 2.15 Data Monitoring Committee and Trial Status; Section 2.13 Adverse Events and Safety Monitoring |
| Methods: Monitoring | Harms and adverse events | All adverse events (AEs) recorded within 24 hours; at least one physician evaluates and treats each AE; serious AEs (SAEs) reported promptly to the ethics committee and relevant authorities; acupuncture-related AEs (e.g., bleeding, dizziness, infection) are described; affected participants may withdraw or discontinue treatment and will be followed up. | Section 2.13 Adverse Events and Safety Monitoring |
| Methods: Monitoring | Auditing | Routine oversight by the institutional ethics committee and hospital; no separate external audit planned; the DMC provides independent monitoring of trial conduct and safety. | Section Ethics and Dissemination  ; Section 2.15 Data Monitoring Committee and Trial Status |

### Ethics and dissemination

| **Section** | **Item** | **How addressed in this protocol** | **Location in this protocol** |
| --- | --- | --- | --- |
| Ethics and dissemination | Ethics approval | States that the trial is conducted in accordance with the Declaration of Helsinki and approved by the Medical Ethics Committee of Shenzhen Hospital (Futian), Guangzhou University of Chinese Medicine; original approval number and amendment approval are reported. | Section Ethics and Dissemination |
| Ethics and dissemination | Protocol amendments | Describes amendment (Protocol Version 1.1) clarifying outcome hierarchy; notes ethics approval date and registry update; states that any future changes to protocol or consent will be submitted to the ethics committee and updated in the registry. | Section 2.1 Registration and Trial Design; Section Section Ethics and Dissemination |
| Ethics and dissemination | Informed consent | All participants (patients and healthy controls) will sign written informed consent prior to enrolment; consent procedures approved by ethics committee; amendment did not alter consent content or process. | Section 2.3 Eligibility Criteria; Section Section Ethics and Dissemination |
| Ethics and dissemination | Consent for publication | States that written informed consent will be obtained for publication of anonymized data and any images that do not contain personally identifiable information. | Data Availability Statement |
| Ethics and dissemination | Confidentiality | Explains that participant data will be stored in a secure database with access limited to authorized study personnel; personal identifiers will be removed before data sharing; de-identified datasets will be shared under data use agreements. | Section 2.2 Study Setting and Recruitment (confidentiality); Section 2.11 Data collection |
| Ethics and dissemination | Declaration of interests | Authors declare no conflicts of interest. | Conflicts of Interest section |
| Ethics and dissemination | Access to data | Specifies that de-identified clinical and neuroimaging datasets will be made available in a public repository after trial completion and publication; analysis scripts can be shared on reasonable request. | Data Availability Statement |
| Ethics and dissemination | Ancillary and post-trial care | Participants continue to receive guideline-based stroke and dysphagia care according to routine practice; management of AEs/SAEs described; no special post-trial interventions or compensation beyond usual care are planned. | Section 2.6 Concomitant therapy; Section 2.13 Adverse Events and Safety Monitoring |
| Ethics and dissemination | Dissemination of results | Trial results (clinical and imaging) will be disseminated through peer-reviewed publications and academic conferences; this protocol itself is submitted for publication. | Abstract; Discussion; Conclusions |
| Ethics and dissemination | Authorship | Authorship and responsibilities are defined using CRediT taxonomy; all authors will approve the final manuscript; no professional medical writers were used. | Author Contributions section |
| Ethics and dissemination | Public access to protocol, dataset, and code | States that the full protocol will be published; de-identified participant-level data and key derived neuroimaging metrics will be shared in a public repository; analysis code will be made available on reasonable request. | Data Availability Statement; Supplementary Materials (SPIRIT Figure and Checklist) |

**Appendices**

| **Section** | **Item** | **How addressed in this protocol** | **Location in this protocol** |
| --- | --- | --- | --- |
| Appendices | Informed consent materials | Notes that the blank informed consent form template approved by the ethics committee will be provided as a confidential supplementary document for editors/reviewers (not publicly posted). | Referenced in Section Ethics and Dissemination |
| Appendices | Biological specimens | No biological specimens are collected, stored, or analysed in this trial; therefore, this item is not applicable. | Not applicable (N/A) – stated in SPIRIT Checklist only |
