# Supplementary material for: Multimodal neuroimaging protocol to explore the neural mechanisms of Tiao Shen Li Yan acupuncture in post-stroke dysphagia: a randomized sham-controlled clinical trial
Source: Front Neurol. 2026 Jun 19;17:1764500. doi: 10.3389/fneur.2026.1764500 (PMC13333810; doi:10.3389/fneur.2026.1764500)
Supplement: Supplementary file 1 [file Supplementary_file_1.zip › supplementary/Supplementary_Material.docx]

Supplementary Material

# Supplementary Data

Not applicable.

# Supplementary Figures and Tables

## Supplementary Figures


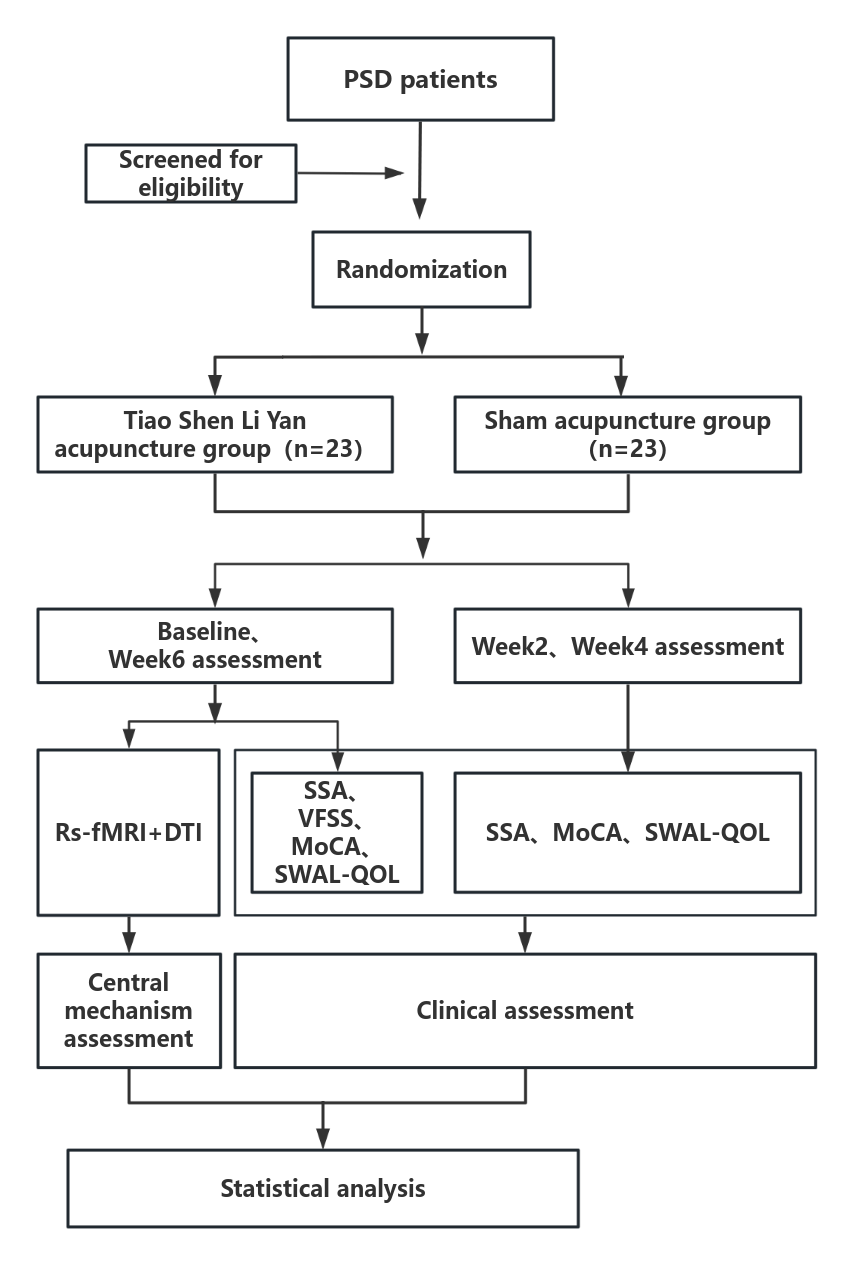


**Figure 1.** Flow chart of the clinical trial procedures.


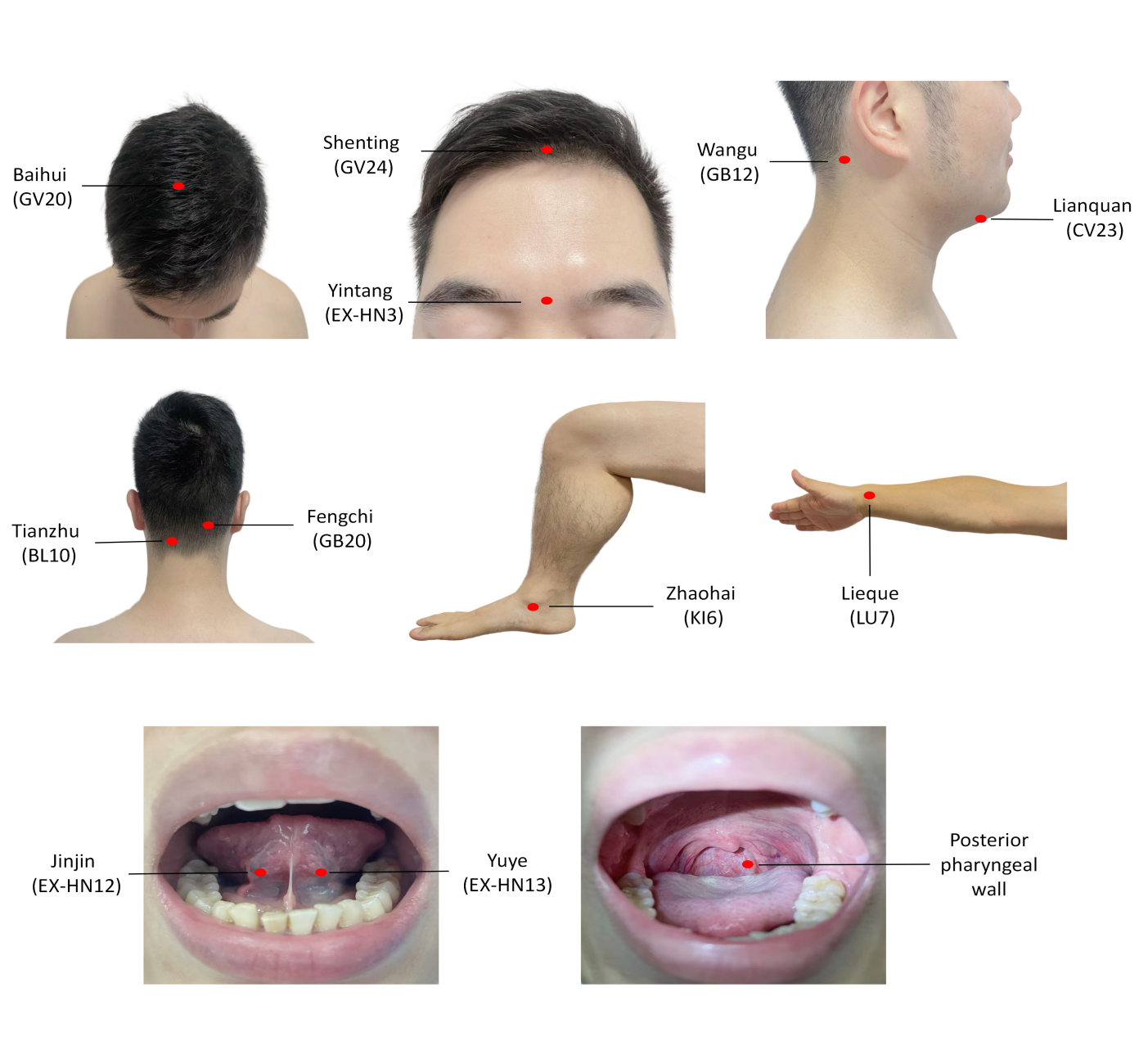


（Artwork by Jing Luo, Qibin Yao）

**Figure 2.** This is an acupoint location figure. Acupoint Location for the Tiao Shen Li Yan acupuncture: Baihui (GV20): Located at the vertex on the midline of the head, where it crosses the line joining the apices of both ears; Shenting (GV24): Located 0.5 cun above the midpoint of Anterior hairline; Yintang (EX-HN3): Located in the middle between the ends of the eyebrows in the glabellar area; Lianquan (CV23): With the head slightly extended, the point lies above the Adam's apple, at the midpoint of the upper border of the hyoid bone body; Fengchi (GB20): Found below the occipital bone, in the depression between the sternocleidomastoid and the superior trapezius muscle; Tianzhu (BL10): Located 0.5 cun above the posterior hairline, 1.3 cun lateral to the midline, in the depression of lateral margin of trapezius muscle; Wangu (GB12): In the hollow just posteroinferior to the mastoid process; Lieque (LU7): Approximately 1.5 cun near the wrist crease, above the styloid process of the radius, in the recess between the tendons of the brachioradialis and abductor pollicis longus muscles; Zhaohai (KI6): Located in the depression below the medial malleolus; Jinjin (EX-HN12): on the left of the lingual frenulum on the sublingual vein of the mouth; Yuye (EX-HN13): on the right side of the lingual frenulum of the oral cavity. Acupoint locations are described according to standard acupuncture textbooks, with minor modifications in wording for clarity.


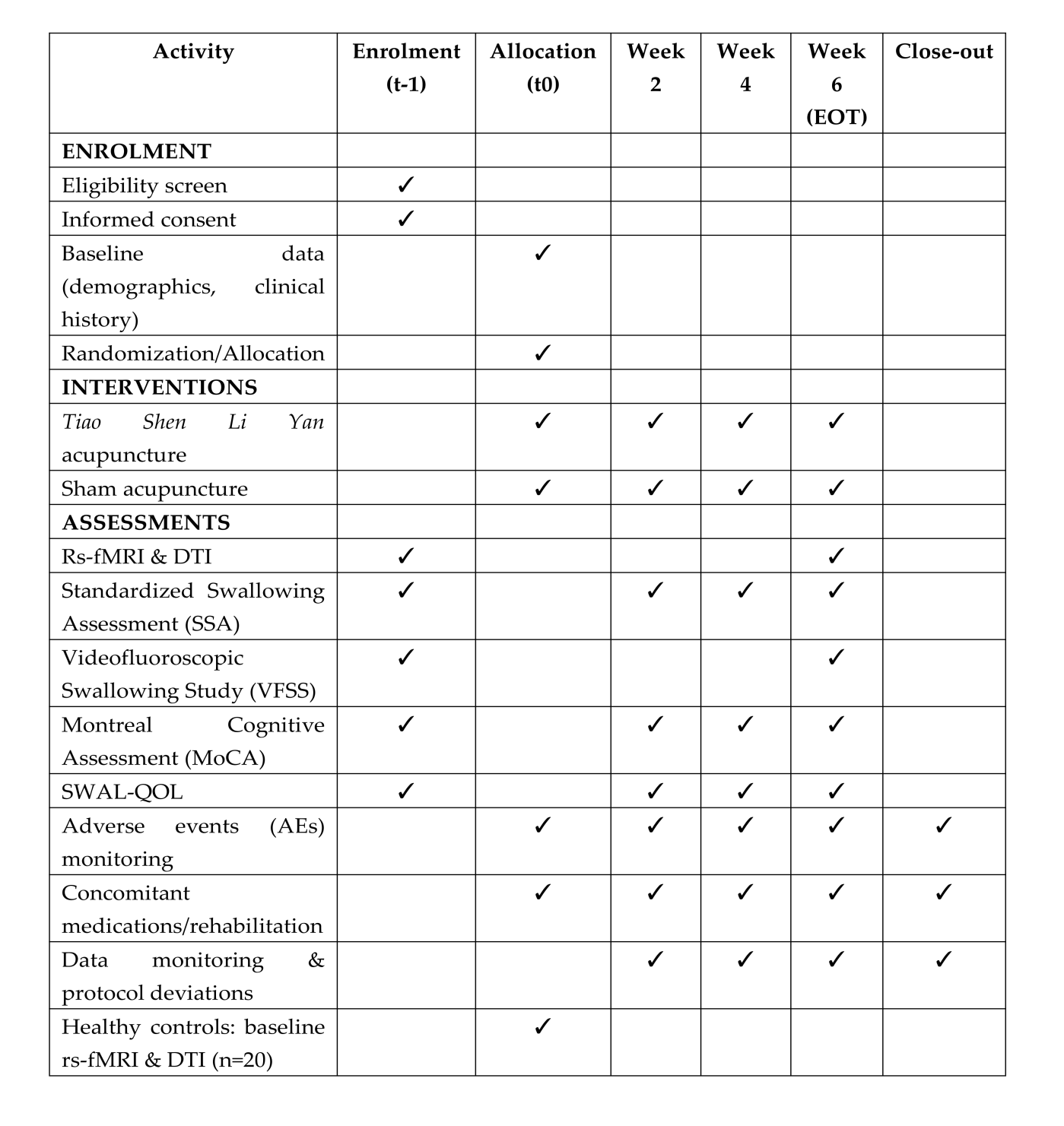


**Figure 3.** SPIRIT schedule of enrolment, interventions, and assessments. Notes: EOT = End of Treatment; rs-fMRI/DTI at baseline (t0) and Week 6; SSA at t0/Weeks 2/4/6; VFSS/MoCA/SWAL-QOL at t0 and Week 6; AEs monitored throughout.

# 2.2 Supplementary Tables

| Assessment | Baseline (W0) | Week 2 | Week 4 | Week 6 |
| --- | --- | --- | --- | --- |
| SSA | ✔ | ✔ | ✔ | ✔ |
| Rs-fMRI and DTI | ✔ |  |  | ✔ |
| VFSS | ✔ |  |  | ✔ |
| MoCA | ✔ | ✔ | ✔ | ✔ |
| SWAL-QOL | ✔ | ✔ | ✔ | ✔ |

Table 1. Assessment schedule.
