# Supplementary figures and images for: Multimodal neuroimaging protocol to explore the neural mechanisms of Tiao Shen Li Yan acupuncture in post-stroke dysphagia: a randomized sham-controlled clinical trial
Source: Front Neurol. 2026 Jun 19;17:1764500. doi: 10.3389/fneur.2026.1764500 (PMC13333810; doi:10.3389/fneur.2026.1764500)

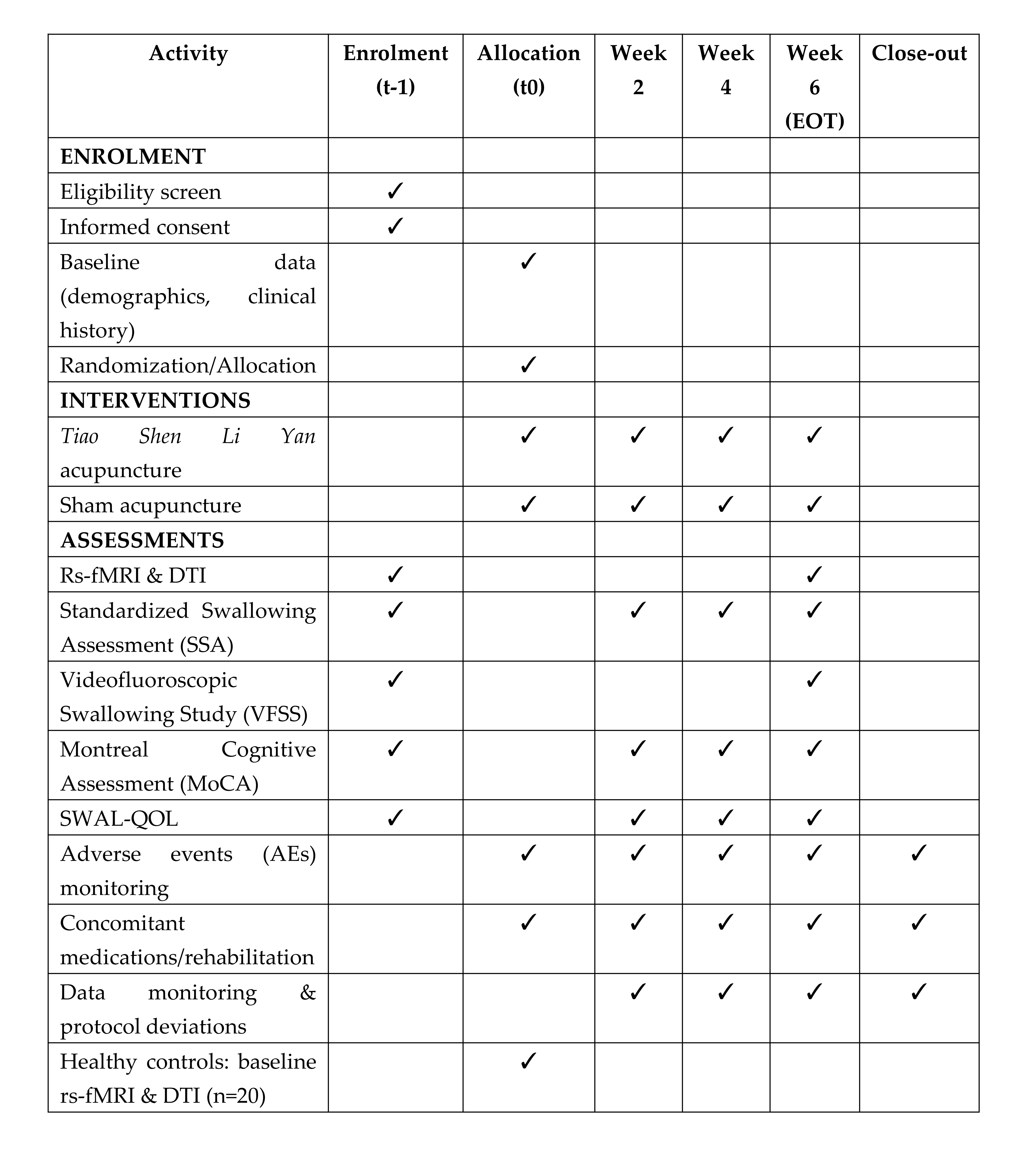

Supplement: Supplementary file 1 [file Supplementary_file_1.zip › supplementary/SPIRIT Figure of enrolment, interventions, and assessments.png]
